# A Standardized Multimodal Neurological Monitoring Protocol-Guided Cerebral Protection Therapy for Venoarterial Extracorporeal Membrane Oxygenation Supported Patients

**DOI:** 10.3389/fmed.2022.922355

**Published:** 2022-06-23

**Authors:** Xiaobei Shi, Qiao Gu, Yiwei Li, Mengyuan Diao, Xin Wen, Wei Hu, Shaosong Xi

**Affiliations:** ^1^Department of Radiology, Affiliated Hangzhou First People's Hospital, Zhejiang University School of Medicine, Hangzhou, China; ^2^Department of Critical Care Medicine, Affiliated Hangzhou First People's Hospital, Zhejiang University School of Medicine, Hangzhou, China

**Keywords:** VA-ECMO, neurologic impairment, multimodal neurological monitoring, protocol, long-term outcomes

## Abstract

**Background:**

The main objective of this study was to investigate the role of a multimodal neurological monitoring (MNM)-guided protocol in the precision identification of neural impairment and long-term neurological outcomes in venoarterial extracorporeal membrane oxygenation (VA-ECMO) supported patients.

**Methods:**

We performed a cohort study that examined adult patients who underwent VA-ECMO support in our center between February 2010 and April 2021. These patients were retrospectively assigned to the “with MNM group” and the “without MNM group” based on the presence or absence of MNM-guided precision management. The differences in ECMO-related characteristics, evaluation indicators (precision, sensitivity, and specificity) of the MNM-guided protocol, and the long-term outcomes of the surviving patients were measured and compared between the two groups.

**Results:**

A total of 63 patients with VA-ECMO support were retrospectively assigned to the without MNM group (*n* = 35) and the with MNM group (*n* = 28). The incidence of neural impairment in the without MNM group was significantly higher than that in the with MNM group (82.1 vs. 54.3%, *P* = 0.020). The MNM group exhibited older median ages [52.5 (39.5, 65.3) vs. 31 (26.5, 48.0), *P* = 0.008], a higher success rate of ECMO weaning (92.8 vs. 71.4%, *P* = 0.047), and a lower median duration of building ECMO [40.0 (35.0, 52.0) vs. 58.0 (48.0, 76.0), *P* = 0.025] and median ECMO duration days [5.0 (4.0, 6.2) vs. 7.0 (5.0, 10.5), *P* = 0.018] than the group without MNM. The MNM-guided protocol exhibited a higher precision rate (82.1 vs. 60.0%), sensitivity (95.7 vs. 78.9%), and specificity (83.3 vs. 37.5%) in identifying neural impairment in VA-ECMO support patients. There were significant differences in the long-term outcomes of survivors at 1, 3 and 6 months after discharge between the two groups (*P* < 0.05). However, the results showed no significant differences in ICU length of stay (LOS), hospital LOS, survival to discharge, or 28-day mortality between the two groups (*P* > 0.05).

**Conclusion:**

The MNM-guided protocol is conducive to guiding intensivists in the improvement of cerebral protection therapy for ECMO-supported patients to detect and treat potential neurologic impairment promptly, and then improving long-term neurological outcomes after discharge.

## Introduction

Some critical patients receive prolonged extracorporeal membrane oxygenation (ECMO) support following the gradual increase in the application of ECMO technology, resulting in the increasing incidence of various complications, which will have a significant impact on the final outcome (1, 2). Central nervous system (CNS) injury is, undoubtedly, a major complication of ECMO, and the causes of such injury vary due to the complexity of ECMO and its invasive nature (3, 4).

According to the Extracorporeal Life Support Organization (ELSO), CNS complications can have a significant impact on long-term survival in ECMO-supported patients (3). In addition, ECMO support during cardiopulmonary resuscitation, named extracorporeal cardiopulmonary resuscitation (ECPR) (5), uses cardiopulmonary bypass to maintain circulatory exhaustion in cardiac arrest (CA) patients in whom traditional CPR is difficult to reverse and has also been increasingly popularized and recognized (6, 7). However, ECPR patients may develop secondary cerebral ischemia and hypoxia during CPR and may receive prolonged cardiopulmonary bypass, therefore, varying degrees of CNS impairment are more likely to occur, such as mild cognitive impairment, cerebral apoplexy, cerebral hemorrhage, ischemic hypoxic encephalopathy, and even brain death (8, 9).

Therefore, the timely identification of a CNS injury by neural monitoring technology during the ECMO support period will contribute to the early initiation of brain protection and intervention therapy, which is conducive to the recovery or alleviation of brain injury. In this case, in the intensive care unit (ICU), multiple neural monitoring modes, such as electrophysiological methods, intracranial pressure measurements, and cerebral oxygenation, are thought to contribute to the prediction and pathophysiological interpretation of brain injury after CA (10).

The operational management of ECMO is complex and challenging. Although most extracorporeal circulation support centers or ICUs have procedures in place to guide their management, CNS injuries that develop in the course of ECMO support are often overlooked. Therefore, the present study aims to guide intensivists with the improvement of cerebral protection therapy for ECMO-supported patients using the multimodal neurological monitoring (MNM) protocol to detect and treat potential neurologic impairment promptly and improve the outcomes of patients.

## Methods

### Study Setting and Population

This study was a cohort study. All data were collected from in-hospital and out-of-hospital adult (>18 years old) patients who underwent venoarterial extracorporeal membrane oxygenation (VA-ECMO) support in Affiliated Hangzhou First People's Hospital, Zhejiang University School of Medicine, between February 2010 and April 2021. This research was reviewed by the Ethics Committee of Affiliated Hangzhou First People's Hospital, Zhejiang University School of Medicine, and the information of relevant patients was anonymously processed to protect privacy.

**Figure 1:**
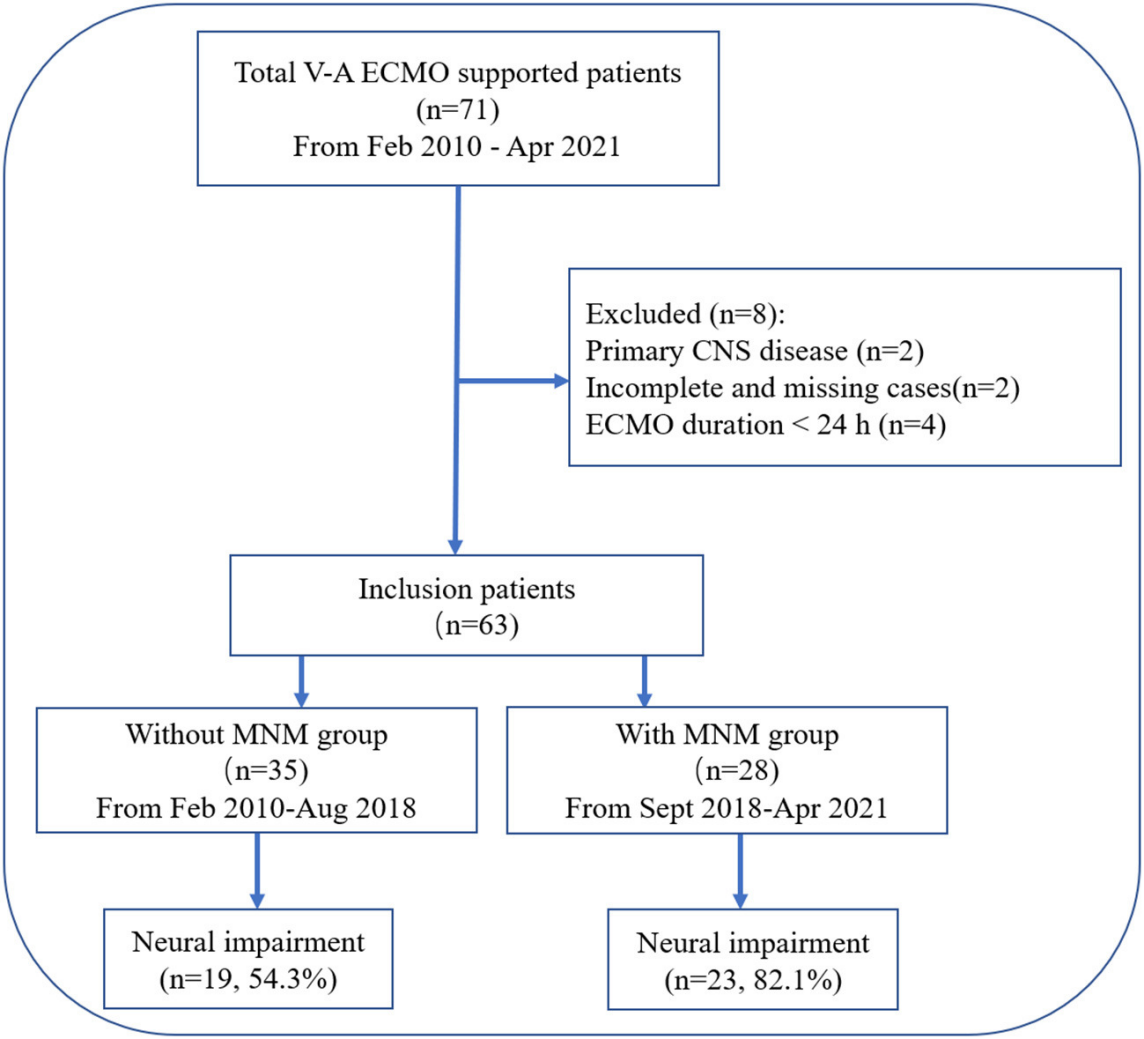
Flow chart of the patient cohort. VA-ECMO, venoarterial extracorporeal membrane oxygenation; CNS, central nervous system; MNM, multimodal neurological monitoring.

Patients admitted from August 2018 underwent daily neurological examinations (physiological and pathological reflexes), and Glasgow Coma Scale (GCS) scores were recorded by two researchers with neurological backgrounds. We established a bedside brain monitoring protocol based on quantitative electroencephalogram (qEEG), transcranial color Doppler ultrasonography (TCCD), and regional cerebral oxygenation (rScO2), named the multimodal neurological monitoring (MNM) protocol (Figure 1), to guide intensivists in the improvement of management practices for ECMO-supported patients and to detect and treat possible neurologic impairment as early as possible. These patients were enrolled in the “With MNM” group.

Patients admitted from February 2010 to August 2018 who underwent traditional and routine ECMO management according to the changes in cardiac function and perfusion indicators (urine volume, lactic acid, mean arterial pressure, etc.) were enrolled in the “Without MNM” group.

Our previous study identified neurological impairment (11): temporal or persistent mental and physical features such as coma, delirium, depression, epilepsy, hypoxic-ischemic encephalopathy, ischemic stroke, cerebral hemorrhage, and death; and a GCS score <15 (patients with endotracheal intubation <11) or cerebral performance category score (CPC) ≥2 after eliminating the disturbance of sedation and analgesia.

Exclusion criteria:

1. Primary CNS disease before admission or previous neuropsychic symptoms.
2. Incomplete and missing cases.
3. Duration of ECMO support <24 h.

### General Management Protocol

**Figure 2:**
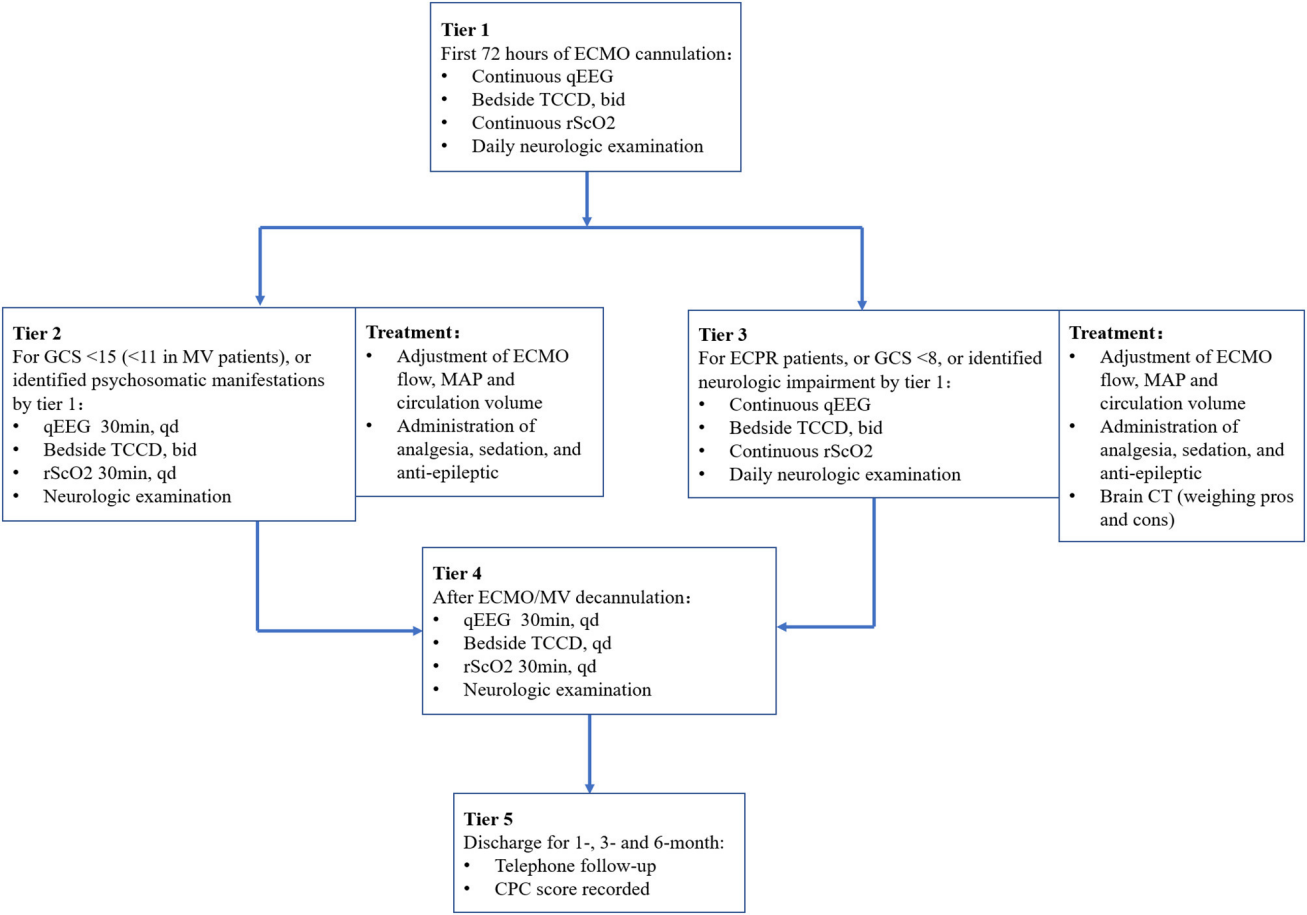
Diagram of the MNM-guided protocol. ECMO, extracorporeal membrane oxygenation; qEEG, quantitative electroencephalogram; TCCD, transcranial color Doppler ultrasonography; rScO_2_, regional cerebral oxygenation; GCS, Glasgow Coma Scale; MAP, mean arterial pressure; CT, computed tomography; MV, mechanical ventilation; CPC, cerebral performance category; bid, bis in die; qd, quaque die.

The MNM-guided protocol included the following (Figure 2): (1) after ECMO, continuous qEEG monitoring and rScO_2_ monitoring were performed. If there were no obvious physical features of neurologic damage within 72 h, the monitoring duration was changed to 30 minutes per day. (2) Bedside TCCD detection was performed twice a day. The blood flow rate and pulse index (PI) of the bilateral middle cerebral arteries (MCAs) were observed, and the bilateral optic nerve sheath diameter (ONSD) was measured to evaluate intracranial blood flow and pressure. (3) Daily neurological examination was performed. (4) Patients underwent ECPR and GCS <8, or after identifying the patients with neurologic impairment through the above procedures, clinicians adjusted the ECMO flow, MAP and circulation volume or administered analgesia, sedation and antiepileptic drugs to maintain the balance among cerebral perfusion, brain oxygen delivery and consumption. For patients with intracranial hematoma, cerebral edema, hypoxic-ischemic encephalopathy and ischemic stroke, clinicians transferred the patients to the imaging center to undergo CT scans to further confirm the diagnosis, and while ensuring patient safety, the next intervention was decided.

### qEEG

qEEG was recorded using the international 10–20 system of electrode placement (NicoletOne Monitor EEG Software, NICVUE 2.9.1, Nicolet, United States) as soon as possible after ECMO support (12, 13). The qEEG was examined by using six electrodes in the left frontal (F3), right frontal (F4), left parietal (P3), and right parietal (P4) positions with ground in the frontal midline and reference in the central midline. The parameters of qEEG included 4 patterns. (1) Amplitude-integrated electroencephalogram (aEEG): aEEG patterns were classified into the following categories to assess cerebral cortex function: continuous normal voltage, discontinuous normal voltage, low voltage, flat, burst suppression, and status epilepticus. (2) Relative Band Power (RBP): RBP could reflect the degree of coma and sleep cycle by directly displaying the percentage of EEG frequencies (δ, θ, α and β) with four kinds of colors (red, yellow, green and blue). (3) Alpha variability: reflects the cerebral blood perfusion and cerebral oxygen metabolism of patients with the patterns of a bar chart. (4) Spectral entropy indicates the depth of sedation or coma. All clinical researchers received special instruction in use of the EEG monitoring operating system. EEG recordings and quantitative EEG trends were reviewed and interpreted by researchers with a professional background in neurology.

### TCCD

According to a previous study (14, 15), bedside TCCD (Philips Ultrasound CX50, Bothell, United States) was performed to insonate the bilateral MCAs and brain parenchyma, and then cerebral flow velocities (CBFVs) and PI values were calculated to assess intracerebral pathology. The quality of the data obtained by TCCD is highly influenced by operator-dependent factors such as skill and experience. The ultrasound operators in the present study completed the training courses of the China Critical Ultrasound Research Group (CCUSG) and obtained qualification certificates.

In addition, the bilateral ONSD was measured by TCCD to reflect intracranial pressure (ICP) (16). The ONSD was measured 3 mm behind the retina and exhibited strong prediction of intracranial hypertension (ICP ≥ 20 mmHg) by a cutoff value of above 5 mm (17).

### rScO_2_

rScO_2_ monitoring by near-infrared spectroscopy (NIRS) offers a validated noninvasive measure of global oxygen delivery and consumption (18, 19). Continuous rScO_2_ monitoring was performed by two NIRS sensors (ECO-N17-C22 L, EnginMed, Suzhou, China) placed on the patients' forehead as soon as possible after ECMO support, and rSO2 values between 55 and 75% were taken as normal rSO2 according to the product manuals. We prefer to record trends of rScO_2_ rather than the absolute values.

### Data Acquisition Outcomes

Patients' baseline characteristics and ECMO-related characteristics were acquired. The primary study outcome was the incidence of neural impairment during ECMO or recovery from ECMO. Secondary outcomes included ICU length of stay (LOS), hospital LOS, 28-day mortality, and neurologic functioning assessed by a CPC score (≥2) at the 1-, 3- and 6-month follow-ups after discharge. The evaluation indicators (precision, sensitivity, and specificity) of the MNM-guided protocol were calculated.

### Statistical Analysis

All data were statistically processed by SPSS 25.0 statistical software. Categorical variables and continuous variables are represented as counts (percentages, %) and medians (interquartile range, IQR). The chi-square test or Fisher's exact test was used for categorical variables, and Student's *t* test or the Mann–Whitney *U* test was used for continuous variables. A *p* < 0.05 was considered statistically significant.

## Results

### Comparison of Baseline Characteristics

After excluding 8 patients, a total of 63 patients with VA-ECMO support were retrospectively assigned to the without MNM group (*n* = 35) and the with MNM group (*n* = 28) based on the presence or absence of the “MNM-guided protocol” (Figure 1).

As shown in Table 1, the incidence of neural impairment in the without MNM group was significantly higher than that in the with MNM group (82.1 vs. 54.3%, *P* = 0.020). However, the results showed no significant differences in ICU LOS, hospital LOS, survival to discharge, or 28-day mortality between the two groups (*P* > 0.05). The results suggested that the MNM-guided protocol could significantly increase the detection rate of neural impairment but did not affect the short-term outcome of patients.

**Table 1:** Comparison of baseline characteristics of patients.

	**All patients**	**MNM protocol**		
	***n* = 63**	**Without (*n* = 35)**	**With (*n* = 28)**	***P***
**Age (years, median)**	40.0 (29.5, 60.5)	31 (26.5, 48.0)	52.5 (39.5, 65.3)	0.008
**Sex**, ***n*** **(%)**				
Male	39 (61.9)	18 (51.4)	21 (75.0)	0.056
Female	24 (38.1)	17 (48.6)	7 (25.0)	
**Underlying diseases**, ***n*** **(%)**				
Hypertension	17 (27.0)	6 (17.1)	11 (39.3)	0.049
Diabetes	8 (12.7)	2 (5.7)	6 (21.4)	0.063
CHD	6 (9.5)	2 (5.7)	4 (14.3)	0.249
**Initiate etiology**, ***n*** **(%)**				
AMI	25 (39.7)	7 (20.0)	18 (64.3)	<0.001
AFM	29 (46.0)	20(57.1)	9 (32.1)	0.048
MA	16 (25.4)	8(22.9)	8 (28.6)	0.605
Others	8 (12.7)	6 (17.1)	2 (7.1)	0.236
**Pre-ECMO characteristics (median)**				
MAP (mmHg)	81.3 (69.5, 94.5)	77.8 (69.3, 87.8)	81.3 (78.3, 109.7)	0.142
CVP (cmH_2_O)	11.0 (8.3, 16.0)	11.0 (8.0, 15.0)	11.0 (9.5, 18.0)	0.197
PH	7.32 (7.22, 7.41)	7.35 (7.28, 7.41)	7.28 (7.17, 7.41)	0.057
PaO_2_ (mmHg)	103.6 (71.6, 207.6)	112.0 (73.4, 192.3)	98.3 (71.1, 209.1)	0.349
PaCO_2_ (mmHg)	37.7 (28.3, 46.9)	37.9 (27.8, 45.0)	37.1 (30.8, 49.3)	0.240
**Other intervenes**, ***n*** **(%)**				
Pre-ECMO PCI	8 (12.7)	7 (20.0)	1 (3.6)	0.052
Post-ECMO PCI	14 (22.2)	1 (2.9)	13 (46.4)	<0.001
IABP	30 (47.6)	15 (42.9)	15 (53.6)	0.397
Pre-ECMO MV	53 (84.1)	27 (77.1)	26 (92.9)	0.090
CRRT	36 (57.1)	17 (48.6)	19 (67.9)	0.124
**Pre-ECMO score (median)**				
SOFA score	11 (8.0, 14.0)	11 (6.0, 14.5)	12 (9.8,13.0)	0.543
APACHE-II score	20 (14.0, 24.0)	18 (11.5, 22.5)	21 (16.0, 26.0)	0.110
**Outcomes (%, median)**				
Neural impairment (%)	42 (66.7)	19 (54.3)	23 (82.1)	0.020
ICU LOS (days)	13.0 (9.0, 21.0)	14.0 (10.0, 19.0)	12.5 (8.8, 22.0)	0.698
Hospital LOS (days)	19.0 (12.5, 26.5)	21.0 (15.0, 26.5)	15.5 (10.8, 25.5)	0.788
Survival to discharge (%)	45 (71.4)	24 (68.6)	21 (75.0)	0.575
28-day mortality (%)	22 (34.9)	13 (37.1)	9 (32.1)	0.679

*MNM, multimodal neurological monitoring; CHD, coronary heart disease; AMI, acute myocardial infarction; AFM, acute fulminant myocarditis; MA, malignant arrhythmia; ECMO, extracorporeal membrane oxygenation; MAP, mean arterial pressure; CVP, central venous pressure; PH, Potential of Hydrogen; PaO_2_, Arterial oxygen partial pressure; PaCO_2_, Arterial carbon dioxide partial pressure; PCI, percutaneous coronary intervention; IABP, intra-aortic balloon pump; MV, mechanical ventilation; CRRT, continuous renal replacement therapy; SOFA, sequential organ failure assessment; APACHE-II, acute physiology and chronic health evaluation II; LOS, length of stay*.

Meanwhile, the MNM group had an older median age [52.5 (39.5, 65.3) vs. 31 (26.5, 48.0), *P* = 0.008], a higher proportion of patients with acute myocardial infarction (64.3 vs. 20.0%, *P* < 0.001), and a higher proportion of patients who underwent percutaneous coronary intervention (PCI) after ECMO support (13 vs. 1%, *P* < 0.001) (Table 1). However, there was no significant difference in some baseline characteristics, laboratory indexes, or critical scores between the two groups.

### The MNM-Guided Protocol Increases the Precision of Identifying Neural Impairment

Of these 63 patients, 42 patients (66.7%) suffered neural impairment, including temporal coma, delirium, seizure, hypoxic-ischemic encephalopathy, intracranial hemorrhage, ischemic stroke, and death (Table 2). As shown in Table 2, in the without MNM group, 13 patients were still in combination with coma or delirium 24–48 h after withdrawal from sedative drugs and were regarded as having neural impairment by initial evaluation. After that, 10 patients without positive physical features were regarded as having delayed awaking resulting from sedative drug accumulation and were excluded after further definite evaluation. Meanwhile, one patient with seizure, one with HIE, one with intracranial hemorrhage and one with ischemic stroke were not identified in the initial assessment. For the patients in the MNM group, 27 patients were regarded as having neural impairment in the initial assessment, exhibited by GCS <15, discontinuous normal voltage and increased δ frequency in aEEG, rScO_2_ <55, MCA-PI increased (>1.05) or decreased (<0.6). Five patients had sedative drug accumulation after further assessment, and one case of ischemic stroke was not identified in the initial assessment. In addition, the MNM-guided protocol exhibited a higher precision rate (82.1 vs. 60.0%), sensitivity (95.7 vs. 78.9%), and specificity (83.3 vs. 37.5%) in identifying neural impairment in VA-ECMO support patients (Table 3).

**Table 2:** Neural impairment in patients with VA-ECMO support.

	**Without MNM (*****n*** **=** **35)**		**With MNM (*****n*** **=** **28)**	
	**Initial**	**Definite**	**Initial**	**Definite**
Coma, *n* (%)	8 (22.9)	1 (2.9)	5 (17.9)	2 (7.1)
Delirium, *n* (%)	5 (14.3)	2 (5.7)	6 (21.4)	4 (14.3)
Seizure, *n* (%)	0 (0.0)	1 (2.9)	3 (10.7)	3 (10.7)
HIE, *n* (%)	1 (2.9)	2 (5.7)	3 (10.7)	3 (10.7)
Intracranial hemorrhage, *n* (%)	0 (0.0)	1 (2.9)	2 (7.1)	2 (7.1)
Ischemic stroke, *n* (%)	0 (0.0)	1 (2.9)	1 (3.6)	2 (7.1)
Total, *n* (%)	14 (40.0)	8 (22.7)	20 (71.4)	16 (57.1)

*MNM, multimodal neurological monitoring; HIE, hypoxic ischemic encephalopathy*.

**Table 3:** Evaluation indicators of MNM-guided protocol.

	**Without MNM (*n* = 35)**	**With MNM (*n* = 28)**
TP (*n*)	15	22
FP (*n*)	10	5
TN (*n*)	6	1
FN (*n*)	4	1
Precision (%)	60.0	82.1
Sensitivity (%)	78.9	95.7
Specificity (%)	37.5	83.3

*MNM, multimodal neurological monitoring; TP, true positive; FP, false positive; TN, true negative; FN, false negative; Precision = TP/(TP + FP); Sensitivity = TP/(TP + FN); Specificity = TN/(FP + TN)*.

These results suggested that the MNM-guided protocol can significantly increase the accuracy of identifying neural impairment in patients supported by VA-ECMO.

### The MNM-Guided Protocol Improves the Precision Management of VA-ECMO-Supported Patients

In the present study, 32 patients (50.8%) underwent ECPR, and there was a significant difference between the “with MNM” and the “without MNM” groups (71.4 vs. 34.3%, *P* = 0.003) (Table 4). In addition, the median duration of building ECMO [40.0 (35.0, 52.0) vs. 58.0 (48.0, 76.0), *P* = 0.025] and median ECMO duration [5.0 (4.0, 6.2) vs. 7.0 (5.0, 10.5), *P* = 0.018] in the with MNM group were significantly lower than those in the without MNM group, and the success rate of ECMO weaning in the with MNM group was significantly higher than that in the without MNM group (92.8 vs. 71.4%, *P* = 0.047) (Table 4).

**Table 4:** Comparison of VA-ECMO related characteristics.

	**All patients**	**MNM protocol**		
	***N* = 63**	**Without (*n* = 35)**	**With (*n* = 28)**	***P***
**ECPR**, ***n*** **(%)**	32 (50.8)	12 (34.3)	20 (71.4)	0.003
**Locations of ECMO**, ***n*** **(%)**				
OR	8 (12.7)	1 (2.8)	7 (25.0)	0.009
ICU	51 (80.9)	30 (85.7)	21 (75.0)	0.282
ED	4 (6.3)	2 (5.7)	2 (7.1)	0.817
**Duration of building ECMO (mins)**	51.0 (40, 63.5)	58.0 (48.0, 76.0)	40.0 (35.0, 52.0)	0.025
**VIS, median**				
Pre-ECMO	20.0 (0, 102.0)	20.0 (0, 55.0)	23.0 (0, 111.3)	0.269
24h post-ECMO	10.0 (0, 18.9)	12.0 (1.5, 20.2)	8.9 (0, 16.5)	0.918
**Continuous NP** **>** **12h**, ***n*** **(%)**	21 (33.3)	14 (40.0)	7 (25.0)	0.209
**ECMO duration (days, median)**	6.0 (5.0, 8.0)	7.0 (5.0, 10.5)	5.0 (4.0, 6.2)	0.018
**MV parameter at 24 h post-ECMO (%, median)**	
FiO_2_ (%)	100 (70, 100)	100 (60, 100)	90 (80, 100)	0.749
PIP (cmH_2_O)	20.0 (16.0, 22.0)	20.0 (16.0, 22.0)	21.0 (17.5, 24.3)	0.476
PEEP (cmH_2_O)	8.0 (7.5, 10.0)	8.0 (6.0, 8.5)	10.0 (8.0, 10.0)	0.036
**Complication**, ***n*** **(%)**			
Cannulation site bleeding	45 (71.4)	25 (71.4)	20 (71.4)	1.000
Limb ischemia	7 (11.1)	5 (14.3)	2 (7.1)	0.370
**ECMO weaning successful**, ***n*** **(%)**	51 (80.9)	25 (71.4)	26 (92.8)	0.047

*MNM, multimodal neurological monitoring; ECPR, extracorporeal cardiopulmonary resuscitation; ECMO, extracorporeal membrane oxygenation; OR, operation room; ED, emergency department; VIS, vasoactive inotropic score [= dose of dopamine + dose of dobutamine + 100 × dose of epinephrine + 10 × dose of milrinone + 10,000 × dose of vasopressin + 100 × dose of norepinephrine (unit: μg/kg/min)]; NP, non-pulsate perfusion; MV, RR, respiratory rate; FiO_2_, fraction of inspiration oxygen; PIP, peak inspiratory pressure; PEEP, positive end expiratory pressure*.

**Figure 3:**
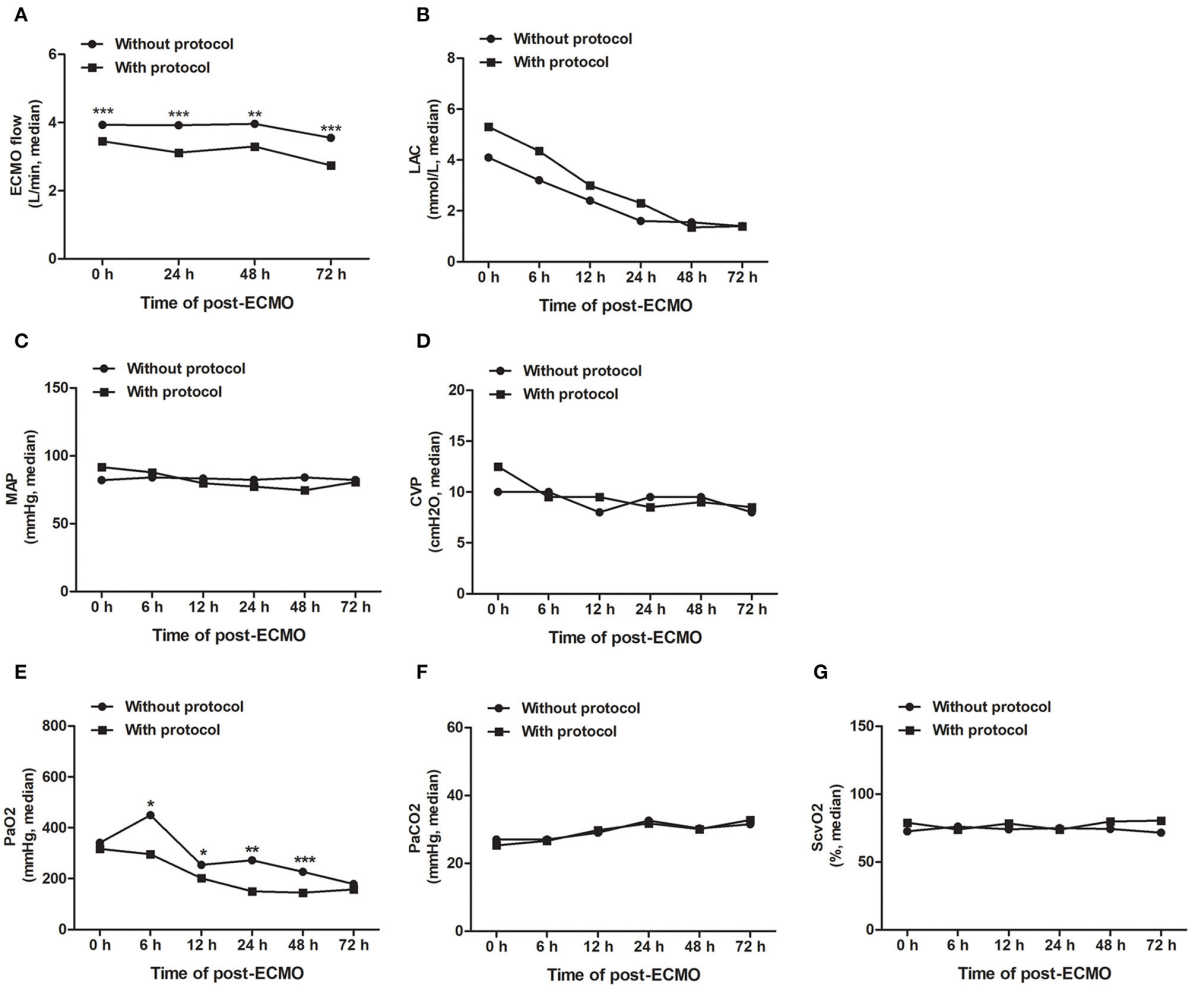
The MNM-guided protocol improves the precision management of VA-ECMO-supported patients. Guided by the MNM protocol, we can accurately adjust the ECMO flow rate **(A)**, maintain appropriate afterload (MAP) and preload (CVP) **(B,C)**, and accelerate the removal of lactic acid **(D)** to achieve the oxygen delivery and consumption balance of patients **(E–G)**. ECMO, extracorporeal membrane oxygenation; MAP, mean arterial pressure; CVP, central venous pressure; ScvO_2_, central venous oxygen saturation; LAC, lactic acid. All data are representative of the median, and the Mann–Whitney U test was used for the comparison. ***P < 0.001, **P < 0.01, *P < 0.05.

As shown in Figure 3, under the guidance of the MNM protocol, we can accurately adjust the ECMO flow rate, maintain appropriate afterload (MAP) and preload (CVP), and accelerate the removal of lactic acid to achieve the oxygen delivery and consumption balance of patients more quickly and to maintain tissue and microcirculation perfusion.

These results suggested that the skill level and operational proficiency of our ECMO team members have advanced considerably in recent years, and the MNM-guided protocol is conducive to the precision management of VA-ECMO-supported patients, helping the early withdrawal of ECMO.

### Long-Term Outcomes of Survivors After Discharge

The long-term adverse neurological outcomes mentioned in this study were identified as neurological complications assessed by a CPC score at the 1-, 3- and 6-month follow-up after discharge, including HIE, stroke, hypomnesia, atypical neuropathy (others), and death (Table 5). As shown in Table 6, 45 patients survived 1 month after discharge, and 3 (14.3%) patients in the MNM protocol group suffered neurological complications, which was significantly lower than that in the without MNM group (11, 45.8%). The MNM group exhibited fewer neurological complications than the without MNM group in surviving patients between 3 months (25.0 vs. 61.9%, *P* = 0.017) and 6 months (15.0 vs. 52.4%, *P* = 0.012) after discharge. These results suggested that the MNM-guided protocol during ECMO support could significantly improve long-term neurological outcomes.

**Table 5:** Long-term neurological outcomes after discharge.

	**1 month (*****n*** **=** **45)**		**3 months (*****n*** **=** **41)**		**6 months (*****n*** **=** **41)**	
	**MNM protocol**		**MNM protocol**		**MNM protocol**	
	**With (21)**	**Without (24)**	**With (20)**	**Without (21)**	**With (20)**	**Without (21)**
**CPC** **≥2**, ***n*** **(%)**	3 (14.3)	11 (45.8)	5 (25.0)	13 (61.9)	3 (15.0)	11 (52.4)
**Neuropathy**, ***n*** **(%)**						
HIE	2 (9.5)	1 (4.2)	0 (0.0)	0 (0.0)	0 (0.0)	0 (0.0)
Stroke	1 (4.8)	3 (12.5)	1 (5.0)	2 (9.5)	1 (5.0)	2 (9.5)
Hypomnesia	0 (0.0)	5 (2.1)	2 (10.0)	5 (23.8)	1 (5.0)	5 (23.8)
Others	0 (0.0)	2 (8.3)	0 (0.0)	3 (14.3)	1 (5.0)	4 (19.0)
**Death**, ***n*** **(%)**	0 (0.0)	0 (0.0)	2 (10.0)	3 (14.3)	0 (0.0)	0 (0.0)

*MNM, multimodal neurological monitoring; CPC, cerebral performance category; HIE, hypoxic ischemic encephalopathy; Others, atypical neuropathy*.

**Table 6:** Effect of MNM protocol on long-term neurological outcomes.

**MNM protocol**	**Neural complications after discharge**, ***n*** **(%)**		
	**1 month (*n* = 45)**	**3 months (*n* = 41)**	**6 months (*n* = 41)**
Without	11 (45.8)	13 (61.9)	11 (52.4)
With	3 (14.3)	5 (25.0)	3 (15.0)
*P*	0.023	0.017	0.012

*MNM, multimodal neurological monitoring*.

## Discussion

Following the gradual increase in the clinical application of ECMO and because of recent advances in managing critical illness, advanced age is not considered a contraindication to temporary mechanical circulatory support (20). In our study, the oldest age of patients receiving ECMO support was 78, and an increasing number of very elderly (>80) patients have exhibited good outcomes after short-term mechanical circulatory support (MCS) (21). Mortality and poor functional outcomes are often induced by neurological injury that results from underlying diseases and from complications associated with ECMO support itself (2, 22). Meanwhile, with the advancement of our management experience and technology, we have gradually expanded the application field of ECMO. For high-risk patients with acute coronary events, we prefer to perform ECMO-assisted percutaneous transluminal coronary intervention. The application of extracorporeal cardiopulmonary resuscitation (ECPR) in cardiac arrest patients is also increasing.

The management of VA-ECMO involves the optimization of ECMO flow, circulation capacity, and MAP. The traditional viewpoint is to maintain myocardium intrinsic contractility and left ventricular ejection with minimal ECMO flow and conservative circulation volume, combined with positive inotropic drugs, while maintaining systemic circulation and end-organ perfusion (23, 24). We have long believed that an ECLS facility should achieve at least 20 cases per year to ensure good and adequate patient outcomes and as an indicator for evaluating an experienced ECLS facility (25). In our opinion, it is more important for the good outcome of patients to strengthen precision management during ECMO support, including the adjustment of anticoagulants, prevention of catheter-related bloodstream infection and lower limb ischemic necrosis, especially the early identification and prevention of CNS complications. Zotzmann et al. (26). found that 10% of ECPR patients suffered intracranial hemorrhage (ICH) by early CT scan, highlighting the importance of standardized neural monitoring, especially in the early stages of ECMO support when neurological examination is limited due to deep sedation (27). However, brain CT scans have poor sensitivity for detecting mental diseases and acute ischemic brain injury, and the transfer of patients with ECMO support between departments inevitably involves high-risk or immediate-threat-of-life situations (28).

In our previous study, 65% of VA-ECMO-supported patients suffered neurological complications, and the identification of neurological complications mainly relied on subjective assessment methods such as GCS, CPC score, confusion assessment method for the ICU (CAM-ICU), and neuroimaging examination (11). To more accurately identify the neurological damage in ECMO-supported patients, we established the MNM-guided protocol, which is based on bedside noninvasive techniques such as qEEG, NIRS, and TCCD, combined with GCS and neurological examination, guiding clinicians to optimize the management of VA-ECMO patients. Our MNM-guided protocol is safe and has no adverse reactions. We hope to promote this multimodal, noninvasive monitoring process in more ECLS facilities, so that it can be accepted as a standard management protocol for ECMO-supported patients.

Several published reports have recommended enhanced bedside noninvasive neural monitoring techniques in patients with extracorporeal circulation support and cardiopulmonary resuscitation (13, 14, 29, 30). However, there are few reports that combine multiple technologies for cross-analysis and then form protocol-based guiding strategies for ECMO management. In the present study, a simplified multiparameter quantitative EEG monitoring device was used, which is easy to operate and intuitive to understand and can be mastered by intensivists after 1–3 months of training.

NIRS measurements of rScO_2_ have been applied to traumatic brain injury (TBI), cardiopulmonary resuscitation, stroke, cerebral hypoxia ischemia, acute coma, cardiac surgery, etc. (31–35). Compared with traditional cerebral oxygenation monitoring tools, it can quickly and effectively identify patients at risk of cerebral hypoxia under different pathophysiological conditions and can noninvasively, real-time and continuously monitor oxygen delivery and evaluate the severity of brain injury and predict outcomes.

However, crucially, the brain should have proper cerebral blood flow. We hope to conveniently monitor the resistance of cerebrovascular, cerebral blood flow velocity, and even whether there is cerebral vasospasm, cerebral edema, cerebral hemorrhage, brain midline shift, etc. We found that continuous non-pulsatile perfusion (NP) >12 h was an independent risk indicator for neurological complications in VA-ECMO-supported patients (11). TCCD is a noninvasive test that uses ultrasonography to estimate CBF. Understanding the changes in CBF is helpful to detect new vascular injury during ECMO support and to optimize cerebral perfusion. TCD and TCCD have been applied to stroke, TBI, hypoxic-ischemic encephalopathy, and neurological monitoring during ECMO support (36–39). Point-of-care ultrasound (PoCUS) visualization management of ECMO patients has played an important role in identifying indications, catheterization, flow adjustment, volume management, cardiac function evaluation, etc.

By integrating and cross-analyzing the data obtained from qEEG, rScO_2_ and TCCD monitoring, we can promptly identify neurologic impairment in ECMO patients, and then guide clinicians to adjust the ECMO flow, circulation volume, vasoactive drug dose, target MAP, and mechanical ventilator parameters to maintain proper cerebral blood flow and cerebral perfusion, thereby improving cerebral protection therapy in a timely manner. This also helps clinicians determine whether it is necessary to take risks and transport patients to imaging centers for further radiological examination. Furthermore, published data have revealed persistent functional deficits associated with ECMO support, and neurologic complications following ECMO are associated with negative impacts on long-term quality of life (40, 41). Consistently, the present study also illustrated a positive association between the MNM-guided protocol and long-term neurological outcomes.

In recent years, clinical decisions driven by machine learning models combined with medical big data have attracted increasing attention. Layering patients offers the opportunity to achieve effective and precision medicine, a key task in personalized healthcare (42). In the field of critical care medicine, the application of big data can provide predictive and prognostic models (43, 44) and the discovery of subgroups or clusters of patients who share similar clinical and/or molecular characteristics (45, 46), physiological waveform analysis of bedside monitors and wearable devices (47, 48). Amorim et al. (49) compared the qEEG reactive machine learning method with expert assessment and indicated that machine learning models utilizing quantitative EEG reactivity data can predict long-term outcomes after cardiac arrest. However, the application of machine learning models based on multimodal big data in the treatment decision-making and prognosis analysis of ECMO patients has rarely been reported. Our team is working with software companies to develop a software platform and database that can integrate medical big data, such as multimodal monitoring and wearable devices, HIS databases, and electronic medical record databases, to assist clinicians in the decision-making process and to manage ECMO patients.

## Limitations

This study has some limitations. First, as a single-center longitudinal observational retrospective study, the sample size was small and less convincing than RCT research. Second, in the process of data collection and analysis, there was a lack of stratified analysis of the effects of mechanical ventilation, intra-aortic balloon pump (IABP) and continuous renal replacement therapy (CRRT) on cerebral blood flow during the ECMO support process, especially the stratified analysis of the effects of pulsatile perfusion and MAP on cerebral perfusion, which may lead to biased results. Rigorous randomized clinical trials or alternative study designs are needed in the future.

## Conclusion

We established a safe and adverse reaction-free MNM-guided protocol in the present study that provides complementarity among multimodal parameters, which is conducive to guiding intensivists in the improvement of cerebral protection therapy for ECMO-supported patients to detect and treat potential neurologic impairment promptly and then improving long-term neurological outcomes after discharge. We hope to promote this multimodal and noninvasive monitoring strategy in more ECLS facilities. In the future, machine learning models combined with multimodal AI big data may help intensivists make clinical decisions, and implement real-time, precision management in ECMO-supported patients.

## Data Availability Statement

The original contributions presented in the study are included in the article/supplementary material, further inquiries can be directed to the corresponding authors.

## Ethics Statement

The studies involving human participants were reviewed and approved by the Ethics Committee of Affiliated Hangzhou First People's Hospital, Zhejiang University School of Medicine. The patients/participants provided their written informed consent to participate in this study.

## Author Contributions

XS, QG, YL, MD, and XW: acquisition and analysis of data. XS: writing of the original manuscript and statistical analysis. XS and SX: revision and editing of the manuscript. MD and WH material, technical, and administrative support and supervision. WH, SX, XS, and QG contributed to study concept and design. All authors approved the final version of the manuscript and agree to be responsible for all aspects of the work.

## Funding

This work was supported by the Zhejiang Provincial Medical and Health Technology Project (Grant No. 2022KY255), and the Construction Fund of Medical Key Disciplines of Hangzhou (2020–2024).

## Conflict of Interest

The authors declare that the research was conducted in the absence of any commercial or financial relationships that could be construed as a potential conflict of interest.

## Publisher's Note

All claims expressed in this article are solely those of the authors and do not necessarily represent those of their affiliated organizations, or those of the publisher, the editors and the reviewers. Any product that may be evaluated in this article, or claim that may be made by its manufacturer, is not guaranteed or endorsed by the publisher.
